# Intimate partner violence and use of reproductive health services among married women: evidence from a national Bangladeshi sample

**DOI:** 10.1186/1471-2458-12-913

**Published:** 2012-10-29

**Authors:** Mosiur Rahman, Keiko Nakamura, Kaoruko Seino, Masashi Kizuki

**Affiliations:** 1 International Health Section, Division of Public Health, Graduate School of Tokyo Medical and Dental University, Yushima 1-5-45, Bunkyo, Tokyo, 113-8519, Japan; 2 Health Promotion, Division of Public Health, Graduate School of Tokyo Medical and Dental University, Tokyo, Japan

**Keywords:** Antenatal care, Delivery assistance, Medical professional, Intimate partner violence, Bangladesh

## Abstract

**Background:**

Data from a statewide survey in India and clinic-based studies in developed settings have previously suggested an association between maternal physical intimate partner violence (IPV) experiences and the low use of antenatal care (ANC). This study aimed to explore the association between maternal experiences of physical and sexual IPV and the use of reproductive health care services, using a large nationally representative data set from Bangladesh.

**Methods:**

This paper used data from the 2007 Bangladesh Demographic Health Survey. The analyses were based on the responses of 2001currently married women living with at least one child younger than 5 years. Exposure was determined from maternal reports of physical and sexual IPV. The utilization of ANC according to amount and type of provider and utilization of delivery assistance according to provider type were used as proxy outcome variables for reproductive health care utilization. Descriptive statistics and multivariate logistic regression analysis used in the study.

**Results:**

Approximately two out of four (48.2%) respondents had experienced physical IPV. Maternal experience of physical IPV was associated with low use of receiving sufficient ANC (adjusted odds ratio [AOR] 0.69; 95% confidence interval [CI] 0.49–0.96), lower likelihood of receiving ANC (AOR 0.69; 95% CI 0.53–0.89), and assisted deliveries from skilled provider (AOR 0.54; 95% CI 0.37–0.78). Women who had been sexually abused were significantly less likely to have visited a skilled ANC and delivery care provider. Furthermore, severity of physical IPV appeared to have more profound consequences on the outcome measured.

**Conclusions:**

The association between exposure to IPV and use of reproductive health care services suggests that partner violence plays a significant role in lower utilization of reproductive health services among women in Bangladesh. Our findings suggest that, in addition to a wide range of socio-demographic factors, preventing maternal physical and sexual IPV need to be considered as an important psychosocial determinates for the higher utilization of reproductive health care services in Bangladesh.

## Background

There has been substantial progress over the past decade in reducing maternal mortality. However, Bangladesh continues to have one of the highest rates. In 2007, maternal mortality ratio (MMR) in Bangladesh was 348 per 100,000 live births contributing to an estimated 3% of the global burden [1]. Despite the wide recognition that two major factors contributing to high maternal mortality are the low use of antenatal care (ANC) and assisted deliveries by non-medically trained personnel [2-5], the proportion of women receiving ANC and assisted deliveries by medically trained personnel (MTP) in Bangladesh remains low. In Bangladesh, 44% of women received no ANC in 2007, and of those who received ANC, only a little more than a quarter completed the minimum of four ANC visits for each pregnancy [6] as recommended by World Health Organization (WHO), to detect health problems associated with pregnancy. The proportion of births attended by medically trained personnel was only about 24.4% [6].

Although service accessibility [7], demographic [8,9], and socio-economic [10-12] risk factors for the low utilization of reproductive health services in Bangladesh are well known, the role that psychosocial factors play in women’s use of reproductive health services is less understood. Intimate partner violence (IPV), which consists of a range of sexually, psychologically, and physically coercive acts perpetrated against women by current or former male intimate partners [13] is considered to be one of the main psychosocial risk factors that might influence use of reproductive health services. A manifestation of Bangladeshi paternalistic culture, where traditional gender paradigms exist, IPV affects 69% of Bangladeshi women during their lifetimes [14]. There are several specific forms of mechanisms through which IPV can affect health service utilization. Evidence shows that victims of IPV have a lower probability of decision making power, decreased freedom of movement and higher economic dependency [15,16], reducing a woman’s ability to make decisions for herself and her family including the choice of receiving appropriate reproductive health care. Victims of IPV are also reported to have social problems and lack of family support including restricted access to services, strained relationship with health providers and employers and isolation from social networks [17]. Furthermore, IPV can lead to inadequate health seeking behavior through different forms of physical, mental, and emotional health impairments such as trauma, walking problems, chronic pain, stress, depression, anxiety, dizziness, functional disorders, and other mental health problems [14,18,19].

Within and outside of South Asia, increasing evidence has shown a linkage between high rates of IPV among women (18-73%) [14,20] and various reproductive health consequences, such as miscarriage [21], preterm labor, low birth weight, stillbirth [22,23], and symptoms of gynecologic morbidity [24]. However, little research has been conducted to examine the association between reproductive health service utilization and IPV. Most studies in this area have been restricted to special populations (e.g., based on clinic-based data) and have been conducted in the developed world [25,26]. However, within South Asia, the only study examining this issue was an investigation in India involving a statewide sample that re-interviewed rural women in four states [27]. These studies examined the association between delayed entry into ANC and physical IPV. However, sexual IPV occurs throughout the world and available data suggest that in many countries including Bangladesh nearly one in four women experience sexual violence by an intimate partner [28-30]. Evidence suggests that sexual IPV has a profound impact on women’s reproductive health consequences, with both immediate and long-term consequences [31,32]. Sexual IPV was found to be associated with significantly increased probability of reporting ever feeling depressed, vaginal bleeding or infection, unwanted pregnancy, pregnancy complications or miscarriages, and lower health service utilization [31-33].

Therefore, there is a need to use nationally representative data to better understand whether physical and sexual IPV are associated with maternal reproductive health service utilization. In the present study, we examined the association between physical and sexual IPV and the utilization of ANC services according to the amount and type of provider as well as the utilization of delivery assistance according to provider type as proxy outcome variables for reproductive health utilization in a nationally representative sample of households in Bangladesh

## Methods

### Data sources

We used data from the 2007 Bangladesh Demographic Health Survey (BDHS), conducted by the National Institute for Population Research and Training of the Ministry of Health and Family Welfare of Bangladesh from March 24 to August 11, 2007. The BDHS sample was drawn from Bangladeshi adults residing in private dwellings. A stratified, multistage cluster sample of 361 primary sampling units was constructed (134 in urban areas and 227 in rural areas). The primary sampling units were derived from a sampling frame created for the 2001 Bangladeshi census.

**Figure 1:**
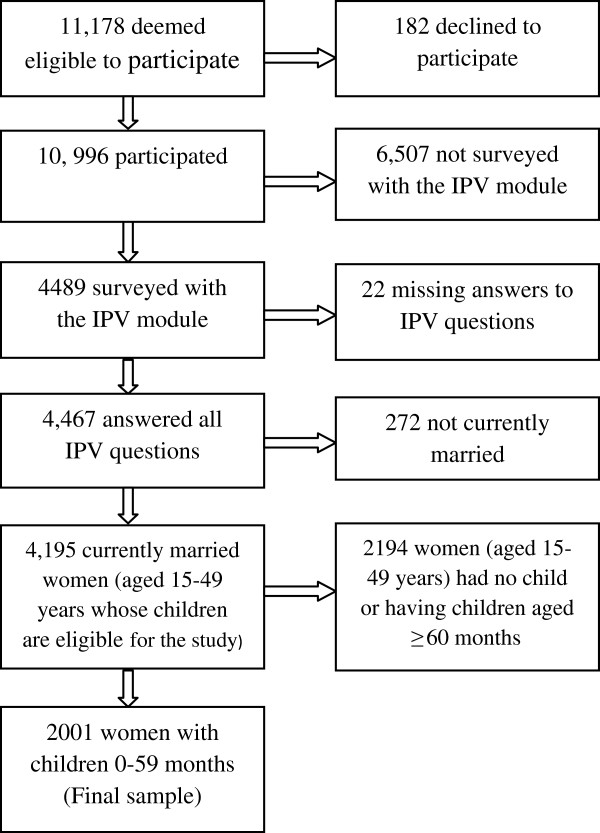
Selection of the sample.

The 2007 BDHS used 5 questionnaires. The questionnaires were drafted in English and then translated into Bangla, the national language of Bangladesh. Of the 11,178 ever-married women aged 15 to 49 years deemed eligible to complete the women’s questionnaire on maternal and child health behaviors and outcomes, 10,996 did so, yielding a response rate of 98.4%. The domestic violence module is a relatively new addition to BDHS and is administered to one, randomly selected, woman by using Kris grid method per household [34]. Of the 4,489 ever–married women aged 15–49 years of age to respond to this module, only 7 had to be excluded because of a lack of privacy. Privacy is important because an abusive husband’s discovery that his wife has disclosed details on IPV to an interviewer could put the woman at risk of further violence. An additional 15 women were not interviewed for other reasons. In our analyses, we included only currently married women aged 15 to 49 years living with at least one child younger than 5 years (n=2001; Figure 1).

### Outcome measures

Utilization of ANC services according to amount and type of provider, and utilization of delivery assistance according to provider type were used as proxy outcome variables of reproductive health care utilization. To assess the amount of ANC services received, a trichotomous categorical variable was created from the continuous measure of the frequency of ANC visits: 1) sufficient ANC (4 visits), 2) insufficient ANC (≤3 visits), 3) no care. In this study, four or more visits were defined as sufficient ANC, in line with the gold standard definition recommended by the WHO [35].

A variable for type of ANC provider was constructed from the combined responses to the question whether the respondent had obtained any advice/treatment according to the particular practitioner consulted. Responses to these questions were organized into three categories: 1) medically trained provider (e.g., qualified doctor, nurse/ midwife/paramedic, family welfare visitors, medical assistant/ sub assistant medical officer/community skilled birth attendant), 2) non-medically trained provider (e.g., health assistant, family welfare assistant/trained and untrained traditional birth attendant, unqualified doctor, and others), or (3) no care. To assess delivery assistance according to the type of provider, a binary variable was constructed from the combined responses to a question regarding whether the respondent had obtained any “advice or treatment” from a medically trained provider or from a non-medically trained provider.

### Intimate partner violence

IPV was defined here as violence experienced by women at the hand of their spouse at that time. In the BDHS, IPV was measured using a shortened and modified version of the Conflict Tactics Scale (CTS-2) which guarantees high reliability and constructive validity [36] and developed in accordance with WHO recommendations for population-based IPV surveillance [37]. The 2007 BDHS stated that to ensure validity and reliability of the data collected on IPV, fieldworkers underwent careful training in different aspects of interview techniques and the questionnaires were pre-tested in pilot studies. Moreover, in order to make valid cross-national comparisons, the questionnaire used to measure IPV in the BDHS include the same criteria and methods in all cultural contexts [6]. IPV was assessed through 8 items pertaining to lifetime perpetration of violence against women at any time from husbands. Physical IPV items were asked before sexual IPV items.

A binary physical IPV variable measured whether the woman’s husband ever performed any of the following acts against her: (1) pushing, shaking, or throwing an object; (2) slapping; (3) pulling hair or twisting an arm; (4) punching or hitting with a fist or something harmful; (5) kicking or dragging; (6) choking or burning; or (7) threatening or attacking with a knife or gun. The Cronbach α for this measure was 0.82. A binary sexual IPV variable assessed whether the woman’s husband had ever physically forced her to have sex or to involuntarily perform sexual acts. A binary variable was created to measure whether a mother reported joint physical and sexual IPV. A composite variable was also created to assess the impact of degree of severity of physical IPV consisted of items from the less severe and severe sections of the CTS-2 physical assault with three categories: (i) none (ii) only less severe physical IPV experienced and (iii) severe physical IPV experienced. Less severe physical IPV was defined when women experienced any of the following options: being ‘pushed’, ‘shaken’, or ‘slapped’. Severe physical IPV was defined when women reported any of the four options: being ‘twisted arm’ ‘burned’ ‘even ‘kicked and punched with fist or something harmful’ or ‘attacked with weapons’.

### Covariates

This study included several socioeconomic and demographic variables that have been theoretically and empirically linked to IPV [20,38] and the utilization of reproductive health care services [8,10-12]. Participants’ age was categorized as follows: 15–24, 25–34, and 35–49 years of age. The women’s and husband’s educational level was defined in terms of the formal education system of Bangladesh: no education (0 year), primary (1–5 years), or secondary or higher (6 years or more). To assess women’s decision-making autonomy, this study determined the number of types of family decisions a woman made alone or jointly, including whether to obtain health care for herself, to obtain health care for her child, to make large purchases, to make household purchases, and to visit her relatives [11].

Maternal occupation was classified according to whether the woman was working or not. Place of residence was categorized as rural versus urban. Religion was categorized as Muslim or non-Muslim. This study classified frequency of mass media exposure, which was found to be a strong predictor of reproductive health service utilization in developing countries into three categories: regular, irregular, or not at all [11,39]. Tertiles were used to classify parity and the total number of household members. A dichotomous variable was created to measure pregnancy intentions for the last birth (intended: live birth wanted at time of conception or unintended: live birth wanted after conception or not wanted at all). The BDHS wealth index was used as a proxy indicator of socioeconomic position and each household was assigned to the poorest, middle, or richest tertile.

### Statistical analyses

This study calculated descriptive statistics for sample’s sociodemographic, IPV, and maternal reproductive health service utilization characteristics. χ^2^ test was used to assess sociodemographic differences in IPV perpetration. In all the analyses, the level of significance was set at *P*<0.05 (2-tailed). 4 fully adjusted models were created to analyze each proxy outcome variable of reproductive health care utilization, with each model containing a different IPV predictor (physical IPV vs. no IPV; sexual IPV vs. no IPV; both physical and sexual IPV vs. no IPV; and the degree of severity of physical IPV vs. no IPV). The multicollinearity of the variables was checked by examining the variance inflation factors (VIFs), which was <2.0. The odds ratios (ORs) were estimated to assess the strength of the associations and used the 95% confidence intervals (CIs) for significance testing. All the covariates were entered simultaneously into the multiple regression models. Analyses were performed using Stata version 11.0 (Stata Corp., College Station, TX, USA). ‘Svy’ commands were used to allow for adjustments for the cluster sampling design, sampling weights, stratification, and the calculation of standard errors. These commands used Taylor series linearization method to estimate confidence intervals around prevalence estimates.

### Ethical considerations

Data collection procedures for the BDHS were approved by the institutional review board of ORC Macro. Several specific protections based on WHO’s ethical and safety recommendations for research on IPV were built into the 2007 BDHS. The protocol of the survey was reviewed and approved by the National Ethics Review Committee of the Bangladesh Ministry of Health and Family Welfare. The questions on IPV were administered to only one eligible respondent per household. Before participating, all the participants were asked to provide verbal informed consent after being read a document emphasizing the voluntary nature of the survey. For the IPV section, the respondents were read an additional statement informing them that the questions to follow could be of a sensitive nature and reassuring them of the confidentiality of their responses. Interviews were conducted under the most private conditions afforded by the environments encountered. If privacy could not be ensured, the interviewers were instructed to skip the module.

## Results

### Socio-demographic profile of the respondents

Nearly half of the respondents (49.1%) were 15 to 24 years old, 26.4% had no education, and 78.3% lived in rural areas (Table 1). About 11.2% had no decision-making autonomy. Regarding their occupation status, 72.4% had no jobs; 91.4% were Muslim.

**Table 1:** **Descriptive statistics**, **according to different forms of IPV experienced by currently married women**: **2007 Bangladesh Demographic Health Survey** (**n**=**2001**)

**Characteristic**	**n (%)**	**Physical IPV %**	**Sexual IPV %**	**Both physical and sexual IPV %**
Maternal age, years				
15-24	888 (49.1)	46.6	18.9	13.6
25-34	893 (40.8)	49.9	17.9	13.7
35-49	220 (10.1)	49.9	20.4	18.7
*P* Value		0.466	0.765	0.240
Maternal education				
No education	559 (26.4)	57.3	21.3	17.9
Primary	608 (30.2)	54.0	18.9	15.1
Secondary and higher	832 (43.4)	38.7	16.9	11.2
Data missing	2			
*P* Value		<0.001	0.312	0.024
Husband’s education				
No education	689 (34.5)	56.1	21.6	17.2
Primary	573 (29.2)	52.7	17.5	14.7
Secondary and higher	737 (36.3)	37.3	16.9	10.8
Data missing	2			
*P* Value		<0.001	0.201	0.031
Maternal decision-making and freedom of movement autonomy, no. of aspects^a^				
0	242 (11.2)	50.9	18.5	13.9
1	139 (6.8)	49.1	27.6	18.4
2	212 (11.7)	48.9	18.0	17.3
3	288 (14.8)	47.0	19.7	14.6
4	336 (16.8)	48.4	20.9	15.4
5	782 (38.6)	47.6	16.0	11.8
Data missing	2			
*P* Value		0.983	0.264	0.479
Respondent employed				
No	1486 (72.4)	46.1	16.9	13.2
Yes	515 (27.6)	53.8	23.4	16.5
*P* Value		0.027	0.018	0.164
Area of residence				
Rural	1290 (78.3)	48.8	19.4	14.5
Urban	711 (21.7)	46.4	16.1	12.9
*P* Value		0.491	0.194	0.473
Religion				
Non-Muslim	176 (8.6)	33.9	13.0	9.0
Muslim	1824 (91.4)	49.6	19.2	14.6
Data missing	1			
*P* Value		<0.001	0.165	0.120
No. of household members (tertiles)				
2-4	680 (30.5)	53.9	17.5	14.0
5-6	764 (36.0)	53.1	19.5	15.2
≥7	557 (33.5)	37.9	18.9	13.1
*P* Value		<0.001	0.697	0.660
Wealth index category				
Poor	861 (45.2)	56.3	21.1	16.8
Middle	348 (16.8)	51.3	20.2	16.4
Rich	792 (38.0)	37.3	15.1	10.0
*P* Value		<0.001	0.045	0.007
Frequency of mass media exposure				
Not at all	959 (46.8)	50.3	19.4	15.2
Irregularly	446 (23.3)	51.7	17.3	11.9
Regularly	596 (29.9)	42.4	18.6	14.3
*P* Value		0.054	0.749	0.467
Parity, no				
1	552 (31.5)	41.5	17.5	12.0
2	546 (26.4)	49.7	18.6	13.7
≥3	903 (42.1)	52.4	19.6	16.0
*P* Value		0.007	0.728	0.191
Pregnancy intentions ^b^				
Unintended	631 (28.6)	53.5	23.8	17.3
Intended	1370 (71.4)	46.1	16.6	12.9
*P* Value		0.012	0.006	0.044
No. of ANC visits				
None	778 (39.1)	54.9	21.9	17.5
Insufficient	785 (40.3)	49.2	17.9	13.5
Sufficient	438 (20.6)	33.7	14.0	9.1
*P* Value		<0.001	0.025	0.005
ANC provider qualification				
None	778 (39.1)	54.8	21.9	17.5
NMTP	186 (9.8)	57.0	20.6	15.6
MTP	1037 (51.1)	41.5	15.7	11.2
Data missing	1			
*P* Value		<0.001	0.019	0.005
Delivery assistance				
NMTP	1577 (81.2)	52.6	19.9	15.4
MTP	423 (18.8)	29.5	13.4	8.7
Data missing	1			
*P* Value		<0.001	0.022	0.015
Prevalence of IPV	2001	48.2	18.7	14.1

*Note*. IPV = intimate partner violence; ANC= antenatal health services; MTP=medically trained provider; NMTP=non–medically trained provider. Numbers are unweighted; percentages are weighted.

^a^Aspects of family decisions a woman participated alone or jointly in making.

^b^ Intended: live birth wanted at time of conception or unintended: live birth wanted after conception or not wanted at all.

*p* <0.05 indicates a significant difference between groups.

From the total sample population, 38% were defined as being rich, approximately 30% watched mass media regularly (watching TV), and 28.6% of the births were unintended. More than one third did not have any ANC (39.1%) and only 20.6% received sufficient ANC; 51.1% and 18.8% received ANC and assisted with delivery from a medically trained provider, respectively.

A substantial percentage (48.2%) reported that they had been exposed to physical IPV; 18.7% indicated that they had experienced sexual IPV and 14.1% indicated that they had experienced both types of IPV (Table 1).

### Differential on IPV victimization with demographics of married Bangladeshi women

The bivariate analyses revealed several significant differences in the prevalence of IPV perpetration across various sociodemographic groups (Table 1). Higher prevalence of physical IPV, sexual IPV, and both physical and sexual IPV were identified among those respondents whose pregnancies were unintended and among women belonging to the poorest category of relative household wealth.

Reports of physical IPV and both type of IPV were significantly more frequent among women with no education than among their counterparts. Muslims and women who did not watch mass media at all were more likely to be physically abused by their husbands than non-Muslim women and women who watched mass media regularly.

Women living in smaller household (2–4 persons) and having parity ≥ 3 were at higher risk for physical IPV relative to women living in larger households and having parity <3. In addition, unemployed women were less likely to report having experienced physical IPV and sexual IPV than employed women. Notably, no differences in any form of IPV experiences were detected across urban or rural residence, decision making autonomy, or maternal age category (Table 1).

### Multivariate analysis

#### Associations between IPV and use of reproductive health services

##### Sufficient ANC

Maternal experiences of physical IPV (adjusted odds ratio [AOR] =0.69; 95% confidence interval [CI] =0.49, 0.96) were associated with a low utilization of sufficient ANC. A significant association was observed between the severe physical IPV and a low utilization of sufficient ANC (AOR =0.48; 95% CI =0.28, 0.80; Table 2).

**Table 2:** Adjusted odds ratios for associations between different forms of maternal IPV and use of reproductive health service for currently married women: 2007 Bangladesh Demographic Health Survey (n=2001)

**IPV measure**	**Adjusted odds ratio** (**95**% **Confidence interval**)		
	**Sufficient ANC visit**	**Type of ANC provider (MTP)**	**Delivery assistance (MTP)**
Physical IPV			
None	1.00	1.00	1.00
Yes	0.69 (0.49-0.96)^c^	0.69 (0.53-0.89)^b^	0.54 (0.37-0.78)^b^
Sexual IPV			
None	1.00	1.00	1.00
Yes	0.71 (0.44-1.16)	0.71 (0.50-0.99)^c^	0.57 (0.33-0.99)^c^
Both physical and sexual IPV			
None	1.00	1.00	1.00
Yes	0.65 (0.38-1.12)	0.71 (0.51-0.99)^c^	0.48 (0.25-0.94)^c^
Forms of physical IPV			
None	1.00	1.00	1.00
Only less severe	0.77 (0.54-1.11)	0.74 (0.55-1.00)	0.58 (0.38-0.88)^b^
Severe	0.48 (0.28-0.80)^b^	0.60 (0.42-0.86)^b^	0.41 (0.23-0.74)^b^

Note. IPV = intimate partner violence; ANC= antenatal health services; MTP=medically trained provider; NMTP=non–medically trained provider. Models were adjusted for maternal age, maternal education, husband’s education, maternal decision-making and freedom of movement autonomy, maternal occupation, residence, religion, frequency of mass media exposure, parity, pregnancy intentions, and wealth index category.

Here a, b, and c indicate p<0.001, p<0.01, and p<0.05, respectively.

##### Type of ANC provider

Maternal experiences of physical IPV (AOR =0.69; 95% CI =0.53, 0.89) were associated with low utilization of receiving ANC from a medically trained provider, as were sexual IPV (AOR=0.71; 95% CI=0.50, 0.99), and both types of IPV (AOR=0.71; 95% CI=0.51, 0.99; Table 2). The experience of severe physical IPV was associated with a low utilization of ANC from a medically trained provider (AOR =0.60; 95% CI =0.42, 0.86).

##### Delivery assistance

Maternal experiences of physical IPV (AOR =0.54; 95% CI =0.37, 0.78) were associated with a low use of medically trained provider for assistance with delivery, as were sexual IPV (AOR=0.48; 95% CI=0.25, 0.94), and both types of IPV (AOR=0.48; 95% CI=0.25, 0.94; Table 2). The experience of less severe (AOR=0.58; 95% CI=0.38, 0.88) or severe physical IPV (AOR=0.41; 95% CI=0.23, 0.74; Table 2) were found to be associated with low use of medically trained provider for delivery assistance.

Additional analyses were performed to determine if an interaction was present between various socio-economic gradients and experience of IPV on women’s reproductive health service utilization. However, no significant interactions were observed between various socio-economic gradients at different levels and IPV on the outcomes that were measured.

##### Use of reproductive health services and other covariates

Receiving sufficient ANC and ANC checkup from medically trained provider were associated with maternal education (secondary and higher), husband’s education (secondary and higher), decision making autonomy (5 aspects), area of residence (urban), rich wealth index score, parity (2 or≥ 3), and frequency of mass media exposure (regular) (Table 3). In addition, a significant association was found between receiving sufficient ANC and maternal age (35–49 years). Maternal age (35–49 years), maternal education (primary or secondary and higher), area of residence (urban), parity (≥3), rich wealth index score, frequency of mass media exposure (regularly), and receiving sufficient ANC were associated with assisted deliveries from medically trained provider.

**Table 3:** **Adjusted odds ratios for associations of physical IPV and all covariates with use of reproductive health service for currently married women**: **2007 Bangladesh Demographic Health Survey** (**n**=**2001**)

**Characteristic**	**Adjusted odds ratio (95% Confidence interval)**		
	**Sufficient ANC visit**	**Type of ANC provider (Medically trained)**	**Delivery assistance (Medically trained)**
Maternal age, years			
15-24	1.00	1.00	1.00
25-34	1.50 (0.99-2.28)	1.12 (0.81-1.55)	2.07 (1.22-3.49)^b^
35-49	2.45 (1.23-4.88)^c^	0.98 (0.59-1.61)	4.08 (1.95-8.54)^a^
Maternal education			
No education	1.00	1.00	1.00
Primary	1.35 (0.76-2.40)	1.44 (1.01-2.05)^c^	2.33 (1.03-5.27)^c^
Secondary and higher	3.0 (1.71-5.26)^a^	2.52 (1.69-3.76)^a^	5.40 (2.45-11.87)^a^
Husband’s education			
No education	1.00	1.00	1.00
Primary	1.13 (0.71-1.80)	0.98 (0.70-1.38)	0.83 (0.47-1.46)
Secondary and higher	1.56 (1.03-2.36)^c^	1.42 (1.00-2.04)^c^	1.80 (1.05-3.08)^c^
Maternal decision-making autonomy, no. of aspects^a^			
0	1.00	1.00	1.00
1	2.09 (0.79-5.51)	2.03 (1.06-3.90)^c^	1.05 (0.45-2.47)
2	2.86 (1.17-7.00)^c^	1.32 (0.69-2.52)	0.47 (0.20-1.07)
3	2.94 (1.22-7.07)^c^	1.51 (0.83-2.73)	0.89 (0.41-1.94)
4	2.22 (0.98-5.04)	1.95 (1.12-3.39)^c^	0.70 (0.35-1.41)
5	3.12 (1.50-6.50)^b^	1.62 (1.02-2.58)^c^	0.71 (0.37-1.36)
Respondent employed			
No	1.00	1.00	1.00
Yes	1.07 (0.71-1.60)	1.03 (0.75-1.41)	1.07 (0.70-1.64)
Area of residence			
Rural	1.00	1.00	1.00
Urban	2.18 (1.51-3.14)^a^	1.61 (1.14-2.26)^b^	1.88 (1.18-3.01)^b^
Religion			
Non-Muslim	1.00	1.00	1.00
Muslim	0.58 (0.31-1.08)	0.61 (0.38-0.98)^c^	0.60 (0.35-1.02)
No. o f household members (tertiles)			
2-4	1.00	1.00	1.00
5-6	1.32 (0.90-1.93)	0.93 (0.68-1.26)	0.90 (0.57-1.42)
≥7	1.21 (0.80-1.82)	0.95 (0.67-1.34)	0.62 (0.38-1.00)
Wealth index category			
Poor	1.00	1.00	1.00
Middle	0.89 (0.52-1.53)	1.12 (0.79-1.60)	0.43 (0.55-1.61)
Rich	1.62 (1.01-2.64)^c^	1.98 (1.34-2.91)^b^	1.27 (1.13-2.88)^c^
Frequency of mass media exposure			
Not at all	1.00	1.00	1.00
Irregularly	1.11 (0.69-1.79)	1.14 (0.82-1.58)	0.81 (0.46-1.40)
Regularly	1.52 (1.00-2.29)^c^	1.77 (1.22-2.57)^b^	1.80 (1.00-3.29)^c^
Parity, no			
1	1.00	1.00	1.00
2	0.61 (0.39-0.93)^c^	0.70 (0.48-1.00)c	0.63 (0.38-1.06)
≥3	0.31 (0.18-0.55)^a^	0.59 (0.37-0.94)^c^	0.23 (0.12-0.42)^a^
Pregnancy intention ^b^			
Unintended	1.00	1.00	1.00
Intended	1.27 (0.88-1.81)	1.15 (0.85-1.56)	0.75 (0.49-1.13)
Sufficient ANC visit			
No	---	---	1.00
Yes			4.47 (3.08 -6.47)^a^
Physical IPV			
No	1.00	1.00	1.00
Yes	0.68 (0.49-0.93)^c^	0.69 (0.53-0.89)^b^	0.54 (0.37-0.78)^b^

Note. IPV = intimate partner violence; ANC= antenatal health services; MTP=medically trained provider; NMTP=non–medically trained provider.

^a^Aspects of family decisions a woman participated alone or jointly in making.

^b^Intended: live birth wanted at time of conception or unintended: live birth wanted after conception or not wanted at all.

Here a, b, and c indicate *p*<0.001, *p*<0.01, and *p*<0.05, respectively.

## Discussion

This is the first study of the relation between IPV and utilization of reproductive health services among Bangladeshi women. Findings from this large representative survey indicate that approximately two out of four (48.2%) married Bangladeshi women experienced physical violence from their husbands. This extremely high lifetime prevalence rate is consistent with WHO Multi-country study on women’s health and domestic violence (life time IPV prevalence over 50%) utilizing data from selected urban and rural areas of Bangladesh, where a modified version of CTS-2 has been used [14] and confirms that IPV is alarmingly commonplace in this impoverished South Asian nation, potentially affecting the health of a majority of married Bangladeshi women.

This study further provide evidence of an association between maternal experiences of physical IPV and the low use of sufficient ANC and ANC checkup, and of delivery care by medical professional. The findings of this study demonstrate that women who have experienced sexual IPV or who have experienced both physical and sexual IPV were significantly less likely to have visited a skilled ANC provider and were less likely to have received delivery care from a medical professional.

Data from a statewide survey in India [27] and clinic-based studies in developed settings [25,26] have previously suggested an association between maternal physical IPV experiences and the low use of ANC, but these studies could not address sexual IPV because that information was not available. In addition to the association between delayed entry to ANC and physical IPV, our results indicate that preventing physical and sexual violence by husbands is an important component of improving the use of reproductive health services in Bangladesh.

Therefore, in addition with physical IPV, it is high time that efforts be undertaken in Bangladesh to prevent sexual IPV. Sexual abuse or rape by an intimate partner is not considered a crime: there is no law against marital rape in most South Asian countries, including Bangladesh. Women married to, or cohabiting with, the perpetrator may not consider forced sex to have been a rape [40,41]. The assumption is that once a woman is wed the husband has the right to unlimited sexual access to his wife. Legislation against forced sex, or rape, within the marriage must not only be promulgated but also implemented.

Another important new finding was that severe physical IPV appeared to have more profound consequences on the outcomes that were measured. Previous studies found that an experience of severe physical IPV was a strong predictor of higher levels of stress and depressive symptoms than those with less severe symptoms [42-44]. The higher levels of stress and emotional impact of severe physical IPV on women can potentially affect their emotional and physical health, and this in turn may lead to a lack of incentive to pursue appropriate care seeking behavior.

The currently identified associations of IPV with low use of sufficient ANC and ANC checkup and delivery assistance from medically trained personnel provide a critical context for the elevated rates of negative pregnancy outcomes and symptoms of gynecologic morbidity demonstrated in previous studies [21-24] among women who have experienced IPV (i.e., the currently documented lower utilization of preventive or curative health services during pregnancy likely relate to an increased risk of negative pregnancy outcomes and symptoms of gynecologic morbidity).

As expected, women with higher education were more likely to receive sufficient ANC and ANC checkup and delivery assistance from medically trained personnel. Possible explanation is that, higher educated women may have a greater decision making power on health related matters and also attach a higher value to the welfare and their health [8]. Further, educated mothers will have more confidence in handling the officials and have the ability and willingness to travel outside the home to seek services [10-12].

The study found that younger mothers (15–24 years) were less likely to seek assistance from medically trained personnel or less likely to receive sufficient ANC compared with older mothers (35–49 years). Older women are generally more experienced and knowledgeable about healthcare services and have higher household decision-making power than younger women, which will improve their likelihood of health service use [8,43]. Rural mothers were less likely to seek care from a medically trained provider and less likely to receive sufficient ANC compared with urban mothers. Other studies also have found a negative relationship with utilization of health services by rural women because of the distances that had to be covered to reach these services [43,44].

The results of this study provide evidence of the association between mothers in the richest bands of wealth and higher likelihood of receiving sufficient ANC and receiving ANC and assisted deliveries from skilled provider. Our finding of the association of richest bands of wealth with the important indicators of reproductive health service utilization is consistent with the findings of previous studies in other developing countries [8,10,45] and provides further evidence that wealth inequality is an important risk factor for lower reproductive health service utilization in Bangladesh.

Exposure to the mass media had a positive effect on receiving sufficient ANC, ANC checkup and assisted deliveries from medically trained personnel. Evidence suggests that mass media are effective in information dissemination, which increases awareness about innovations, and fosters inter-personnel communication, which could facilitate behavioral changes allowing for the adoption of new/different behaviors [11,46]. Having more children may also cause resource constraints, which has a negative effect on healthcare utilization. The present results also support the hypothesis indicating that women with more children were less likely to receive sufficient ANC or assisted deliveries from medically trained personnel than women with fewer children [11,47].

The observation that women who received sufficient ANC sought assistance more from medically trained personnel may be due to the fact that ANC may raise awareness of the need for care at delivery, or may give women and their families a familiarity with health facilities that enables then to seek care more efficiently during a crisis. Different studies have found that lack of ANC is an important risk factor for low use of delivery assistance from skilled health personnel [11,46]. Consistent with previous studies, carried out in several developing countries, we found that women’s lack of decision making autonomy is a serious constraint to receiving needed care [11,43,48].

The study therefore suggests that there are several socio-demographic factors interplaying which lead to low use of reproductive health services in Bangladesh. Furthermore consistent with prior research conducted in developing nations including Bangladesh, this study found that low utilization of reproductive health care services were more common among the most disadvantaged strata of Bangladeshi society.

### Strengths and limitations

The main strength of this study is that the data came from a large government survey, carried out in 2007. A relevant subset was extracted consisting of currently married women aged 15–49 years who had a live birth in the 5 years preceding the survey, giving a large sample size of 2001. The BDHS uses extensive interviewer training, standardized measurement tools and techniques, an identical core questionnaire, and instrument pretesting to ensure standardization and comparability across diverse sites and time [6]. In addition, BDHS used behaviorally specific questions to ascertain physical and sexual IPV, and these types of questions are considered the best methodological means for eliciting accurate response [36,49].

Some limitations should be considered when interpreting our findings. First, our analyses were cross sectional, thus, we were unable to determine the exact temporal relationship between IPV and the use of reproductive health care within the limited timeframe. Second, although psychological violence is an important facet of IPV [19], information on this form of violence was not available from the BDHS. Third, because our selection of variables was constrained by the preexisting BDHS data, we were unable to include additional, potentially important variables. Finally, the possibility of underreporting must also be considered; because IPV is by nature a private phenomenon and one that is often stigmatized, women may be reluctant to reveal information. However, the BDHS interviewers were provided with training to implement the domestic violence module based on a training manual specially developed so the field staff could collect violence data in a secure, confidential, and ethical manner that would create a safe atmosphere for respondents to comfortably discuss this issue [6].

## Conclusions

In conclusion, IPV was associated with the low use of reproductive health services among women in Bangladesh. Our findings suggest that, in addition to a wide range of socio-demographic factors, preventing maternal physical and sexual IPV need to be considered as an important psychosocial determinates for the higher utilization of reproductive health care services in Bangladesh. However, future longitudinal studies are needed to investigate the influence of potential mechanisms mediating the association between IPV and the use of reproductive health services.

## Competing interests

The authors declare that they have no competing interests.

## Authors’ contributions

MR originated the study and contributed to study design, statistical analysis, and the writing of the article. KN conceptualized the aim of the study and contributed to study design, statistical analysis, interpretation of the data, and critically revisions of the article. KS and MK contributed to analysis and interpretation of data and to revisions of the article. All authors read and approved the final manuscript.

## Sources of funding

The authors have indicated they have no financial relationships relevant to this article to disclose.

## Pre-publication history

The pre-publication history for this paper can be accessed here:

http://www.biomedcentral.com/1471-2458/12/913/prepub

## References

[B1] BBS Millennium Development Goals: Bangladesh Progress at a Glance 2007 Dhaka: Bangladesh Bureau of Statistics

[B2] Chaudhury RH Multi-sectoral determinants of maternal mortality in Bangladesh 2008 Hefei, Anhui Province, China: Workshop on addressing multi-sectoral determinants of maternal mortality ESCAP Region

[B3] Chowdhury AMR Mahbub A Chowdhury AS Skilled attendance at delivery in Bangladesh: an ethnographic study 2008 BRAC Dhaka, Bangladesh: In Research Monograph Series, Research and Evaluation Division

[B4] Kidney E Winter HR Khan KS Gülmezoglu MA Meads CA Deeks JJ Christine M Systematic review of effect of community-level interventions to reduce maternal mortality BMC Pregnancy Childbirth 2009 9 2 10.1186/1471-2393-9-2 19154588 PMC2637835

[B5] Ronsmans C Scott S Qomariyah SN Achadi E Braunholtz D Marshall T Pambudib E Witten KH Graham WJ Professional assistance during birth and maternal mortality in two Indonesian districts Bull World Health Organ 2009 87 405 484 10.2471/BLT.08.051581 PMC2686212 19565119

[B6] NIPORT, Mitra and Associates, and Macro International Bangladesh Demographic and Health Survey 2007 2009 Dhaka, Bangladesh and Calverton, Maryland, USA: National Institute of Population Research and Training, Mitra and Associates, and Macro International

[B7] Rahman MH Mosley WH Ahmed S Akhter HH Does service accessibility reduce socioeconomic differentials in maternal care seeking? Evidence from rural Bangladesh J Biosoc Sci 2008 40 19 33 17588280 10.1017/S0021932007002258

[B8] Rahman M Haque SE Zahan MS Factors affecting the utilization of postpartum care among young mothers in Bangladesh Health Soc Care Community 2011 19 138 147 20880103 10.1111/j.1365-2524.2010.00953.x

[B9] Chowdhury AMR Mahbub A Chowdhury AS Skilled attendance at delivery in Bangladesh: an ethnographic study 2003 Research and Evaluation Division,BRAC Dhaka, Bangladesh: In Research Monograph Series

[B10] Amin R Shah M Becker S Socioeconomic factors differentiating maternal and child health-seeking behavior in rural Bangladesh: A cross-sectional analysis Int J Equity Health 2010 9 9 10.1186/1475-9276-9-9 20361875 PMC2859349

[B11] Haque SE Rahman M Mostofa MG Zahan MS Reproductive health care utilization among young mothers in Bangladesh: does autonomy matter? Women’s Health Issues 2012 22 171 180 10.1016/j.whi.2011.08.004 21968029

[B12] Paul BK Rumsey DJ Utilization of health facilities and trained birth attendants for childbirth in rural Bangladesh: an empirical study Soc Sci Med 2002 54 1755 1765 10.1016/S0277-9536(01)00148-4 12113433

[B13] WHO World report on violence 1997 Geneva: World Health Organization

[B14] Ellsberg M Jansen HA Heise L Watts CH Garcia-Moreno C Intimate partner violence and women’s physical and mental health in the WHO multi-country study on women’s health and domestic violence: an observational study Lancet 2008 371 1165 1172 10.1016/S0140-6736(08)60522-X 18395577

[B15] Ellsberg M Pena R Herrera A Liljest J Winkvist A Candies in hell: women’s experiences of violence in Nicaragua Soc Sci Med 2000 51 1595 1610 10.1016/S0277-9536(00)00056-3 11072881

[B16] Smith MD Martin F Domestic violence: recognition, intervention, and prevention Med Surg Nurs 1995 4 21 25 7874217

[B17] Heise L Garcia-Moreno C Krug E, Dahlberg LL, Mercy JA Violence by intimate partners World report on violence and health 2002 Geneva (Switzerland): World Health organization

[B18] Ellsberg MC Violence against women: a global public health crisis Scand J Public Health 2006 34 1 4 10.1080/14034940500494941 16449037

[B19] Coker AL Smith PH Bethea L King MR McKeown RE Physical health consequences of physical and psychological intimate partner violence Arch Fam Med 2000 9 451 457 10.1001/archfami.9.5.451 10810951

[B20] Bates LM Schuler SR Islam F Islam MK Socioeconomic factors and processes associated with domestic violence in rural Bangladesh Int Fam Plan Perspect 2004 30 190 199 10.1363/3019004 15590385

[B21] Silverman JG Gupta J Decker MR Kapur N Raj A Intimate partner violence and unwanted pregnancy, miscarriage, induced abortion, and stillbirth among a national sample of Bangladeshi women BJOG 2007 114 1246 1252 10.1111/j.1471-0528.2007.01481.x 17877676

[B22] Jasinski JL Pregnancy and domestic violence: a review of the literature Trauma Violence Abuse 2004 5 47 64 10.1177/1524838003259322 15006296

[B23] Jacoby M Gorenflo D Black E Wunderlich C Eyler AE Rapid repeat pregnancy and experiences of interpersonal violence among low-income adolescents Am J Prev Med 1999 16 318 321 10.1016/S0749-3797(99)00029-X 10493289

[B24] Koenig SA Ahmed S Domestic Violence and Symptoms of Gynecologic Morbidity among Women in North India Int Fam Plan Perspect 2006 32 201 220 10.1363/3220106 17237017

[B25] McFarlane J Parker B Soeken K Bullock L Assessing for abuse during pregnancy: Severity and frequency of injuries and associated entry into prenatal care JAMA 1992 267 3176 3178 10.1001/jama.1992.03480230068030 1593739

[B26] Bailey BA Daugherty RA Intimate partner violence during pregnancy: incidence and associated health behaviors in a rural population Matern Child Health J 2007 11 495 503 10.1007/s10995-007-0191-6 17323125

[B27] Alissa DK Stephenson R Koenig MR Physical violence by partner during pregnancy and use of prenatal care in rural India J Health Popul Nutr 2011 29 245 254 21766560 10.3329/jhpn.v29i3.7872 PMC3131125

[B28] Hadi A Prevalence and correlates of the risk of marital sexual violence in Bangladesh J Interpers Violence 2000 15 787 805 10.1177/088626000015008001

[B29] Kapadia MZ Saleem S Karim MS The hidden figure: sexual intimate partner violence among Pakistani women Eur J Public Health 2010 20 164 168 10.1093/eurpub/ckp110 19666702

[B30] Campbell J Health consequences of intimate partner violence Lancet 2002 359 1331 1336 10.1016/S0140-6736(02)08336-8 11965295

[B31] Holmes MM Resnick HS Kilpatrick DG Best CL Rape-related pregnancy: estimates and descriptive characteristics from a national sample of women Am J Obstet Gynecol 1996 175 320 324 10.1016/S0002-9378(96)70141-2 8765248

[B32] Letourneau EJ Holmes M Chasendunn-Roark J Gynecologic health consequences to victims of interpersonal violence Women’s Health Issues 1999 9 115 120 10.1016/S1049-3867(98)00031-0 10189822

[B33] Leserman J Li Z Drossman DA Hu YJ Selected symptoms associated with sexual and physical abuse among female patients with gastrointestinal disorders: the impact on subsequent health care visits Psychol Med 1998 28 417 425 10.1017/S0033291797006508 9572098

[B34] ICF Macro Demographic and Health Survey Interviewer’s Manual. MEASURE DHS Basic Documentation No. 2 2009 Calverton, Maryland, U.S.A: ICF Macro

[B35] WHO Provision of effective antenatal care: Integrated Management of Pregnancy and Child Birth (IMPAC) 2006 Geneva, Switzerland: Standards for Maternal and Neo natal care (1.6), Department of Making Pregnancy Safer Available: http://www.who.int/making pregnancy safer/publications/Standards1.6N.pdf. Accessed 2009 September 3

[B36] Straus M Hamby S Boney-McCoy S Sugarman D The revised Conflict Tactics Scales (CTS2): development and preliminary psychometric data J Family Issues 1996 17 283 316 10.1177/019251396017003001

[B37] WHO: Putting women first Ethical and safety recommendations for research on domestic violence against women 2001 Geneva: Department of Gender and Women’s Health, World Health Organization

[B38] Uthman OA Lawoko S Moradi T Factors associated with attitudes towards intimate partner violence against women: a comparative analysis of 17 sub- Saharan countries BMC Int Health Hum Rights 2009 9 14 10.1186/1472-698X-9-14 19619299 PMC2718859

[B39] Rahman M Deliveries among the adolescent mothers in rural Bangladesh: who provide assistance? World Health Population 2009 11 5 14 20057269 10.12927/whp.2009.21039

[B40] Heidi B Ruchira J Naved T Spousal violence in Bangladesh: A call for a public-health response J Health Popul Nutr 2008 26 366 377 18831231 PMC2740706

[B41] UNICEF Domestic violence against women and girls .Innocent Digest 2000 Florence-Italy: United Nations Children emergency Fund, Innocent research Center

[B42] Beth AB Partner violence during pregnancy: prevalence, effects, screening, and management Int J Women’s Health 2010 2 183 197 10.2147/ijwh.s8632 PMC2971723 21072311

[B43] Bogat GA Levendosky AA DeJonghe E Davidson WS Von EA Pathways of suffering: The temporal effects of domestic violence on women’s mental health Maltrattamentoe abuso all’infanzia 2004 6 97 112

[B44] Vogel LC Marshall LL PTSD symptoms and partner abuse: Low income women at risk Journal of Posttraumatic Stress 2001 14 569 584 10.1023/A:1011116824613 11534886

[B45] Bloom SS Wypij D Gupta M Dimensions of women’s autonomy and the influence on Maternal Health Care Utilization in a North Indian City Demography 2001 38 67 78 10.1353/dem.2001.0001 11227846

[B46] Rahman M Islam I Islam AZ Rural–urban differentials of utilization of ante-natal health-care services in Bangladesh Health Policy and Development 2008 6 117 125

[B47] Rahman M Haque SE Mostofa MG Tarivonda L Shuaib M Wealth inequality and utilization of reproductive health services in the Republic of Vanuatu: insights from the multiple indicator cluster survey, 2007 Int J Equity Health 2011 10 58 10.1186/1475-9276-10-58 22132828 PMC3286423

[B48] Sharma SK Sawangdee Y Sirirassamee B Access to health: women’s status and utilization of maternal health services in Nepal J Biosoc Sci 2007 39 671 692 10.1017/S0021932007001952 17359562

[B49] Pebley AR Goldman N Rodriguez G Prenatal and delivery care and childhood immunization in Guatemala: Do family and community matter? Demography 1996 33 231 247 10.2307/2061874 8827167

